# Therapeutic potential of a psychosocial intervention in the mental health of women deprived of liberty

**DOI:** 10.1590/0034-7167-2025-0348

**Published:** 2026-09-18

**Authors:** Maria Theresa Veloso Figueiredo de Carvalho, Jaqueline Lemos de Oliveira, Janaína Cristina Pasquini de Almeida, Antonio José Correa de Pauli, Bianca Moretti Tomiazzi, Jacqueline de Souza

**Affiliations:** I Universidade de São Paulo. Ribeirão Preto, São Paulo, Brazil; II Universidade de São Paulo. São Paulo, São Paulo, Brazil

**Keywords:** Psychosocial Intervention, Mental Health, Women, Prisoners, Nursing., Intervención Psicosocial, Salud Mental, Mujeres, Prisioneros, Enfermería.

## Abstract

**Objectives:**

to analyze the therapeutic potential of a psychosocial intervention protocol developed and administered by nurses.

**Methods:**

a pilot, quantitative, preand post-test study, approved by the Ethics Committee and conducted with 12 women deprived of liberty in a women’s penitentiary complex in a municipality in Minas Gerais. Sociodemographic data and data regarding symptoms of common mental disorders and mood modulation were collected. Descriptive statistics, measures of dispersion, and non-parametric tests were used for data analysis.

**Results:**

nine women completed the proposed program. Improvement was identified in all domains analyzed in relation to mood modulation, with particular emphasis on the depression and tension domains. Regarding symptoms of common mental disorders, there was a reduction in descriptive terms, but without statistical significance.

**Conclusions:**

the results suggest that the proposed intervention has the potential to be considered as a non-pharmacological therapeutic option by nurses working in the prison system.

## RESUMEN

**Objetivos:**

analizar el potencial terapéutico de un protocolo de intervención psicosocial desarrollado e implementado por enfermeras.

**Métodos:**

estudio piloto cuantitativo, prey post-prueba, aprobado por el Comité de Ética, realizado con 12 mujeres privadas de libertad en un Complejo Penitenciario Femenino de un municipio de Minas Gerais. Se recopilaron datos sociodemográficos, así como datos sobre síntomas de trastornos mentales comunes y modulación del estado de ánimo. Para el análisis de datos se utilizaron estadísticas descriptivas, medidas de dispersión y pruebas no paramétricas.

**Resultados:**

nueve mujeres completaron el programa propuesto. Se identificó una mejora en todos los dominios analizados en relación con la modulación del estado de ánimo, con énfasis en los dominios de depresión y tensión. En cuanto a los síntomas de trastornos mentales comunes, se observó una reducción en los términos descriptivos, pero sin significación estadística.

**Conclusiones:**

los resultados sugieren que la intervención propuesta tiene el potencial de ser considerada como una opción terapéutica no farmacológica por el personal de enfermería que trabaja en el sistema penitenciario.

## INTRODUCTION

In Brazil, the number of people deprived of their liberty is 500,381, of which 31,181 are women^(1)^. Only 7% of Brazilian prison units are exclusively for women^(2)^, although the Penal Execution Law (Law 7,210 of July 11, 1984) and the Brazilian National Policy for Attention to Women in Situations of Deprivation of Liberty and Released from the Prison System provide for specific prison units for the female public, considering the situations of exclusion and violation of rights frequently experienced by women in mixed prison units^(3-6)^.

The disparity found in the treatment offered to women deprived of their liberty reproduces the inequalities experienced in society in general and exposes the male bias that permeates the rules applied to these women, ignoring the particularities that intersect them such as class, culture, and gender, which results in their marginalization and subjugation^(7)^. Several researchers have dedicated themselves specifically to the female population deprived of liberty. They emphasize the need for healthcare teams to prioritize this population, considering history of trauma, abuse, and neglect generally suffered throughout their lives, associated with the hostile prison environment and human rights violations, such as overcrowding and difficulty accessing healthcare and treatment, as well as basic hygiene items^(3-6)^.

Studies show that such vulnerability factors contribute to the intensification or development of mental disorders, and in some cases culminate in self-destructive behaviors^(4-6,8-11)^. Research conducted with women deprived of liberty in Ceará identified a prevalence of 68.4% of common mental disorders^(4)^, while a study conducted in a penitentiary in Pará revealed that 50% presented symptoms indicative of anxiety, depression and/or stress^(5)^. Internationally, investigations conducted in prisons in Norway and the United Kingdom have indicated prevalence rates of psychiatric disorders of 56% and 76%, respectively^(9,10)^. Furthermore, a systematic review indicated post-traumatic stress disorder as the most frequent mental health problem among incarcerated women^(11)^. These findings, coupled with social stigma and discontinuity of mental healthcare, reinforce the need for mental health to be treated as a priority in prison healthcare policies and practices, with a specific focus on the needs of women deprived of their liberty.

However, access to healthcare within the prison system needs to be accessible and provided under conditions similar to those offered to the general population, even after the implementation of the Brazilian National Policy for Comprehensive Healthcare for People Deprived of Liberty in the Prison System (In Portuguese, *Política Nacional de Atenção Integral à Saúde das Pessoas Privadas de Liberdade no Sistema Prisional* - PNAISP)^(12)^ in 2014, which aims to guarantee the right to health for all incarcerated individuals in the prison system through access to the Brazilian Unified Health System. Among the professionals who make up the minimum team established by the PNAISP, nurses stand out as an extremely important professional for mental healthcare in prisons, since, by considering comprehensive and humanized care, they enable assistance consistent with the real health needs of this population, minimizing the effects of negligence and providing well-being, restoring a dignified life condition and promoting mental health, as pointed out in previous studies^(13-15)^.

Regarding mental health interventions for the population deprived of liberty, studies that used strategies based on Cognitive-Behavioral Theory^(16)^, drama therapy^(10)^, horticultural therapy^(17)^ and support^(18)^ identified positive results for improving self-control, emotional regulation, self-esteem, life satisfaction, subjective well-being and the development of social skills^(10,16-18)^. These findings highlight the importance of considering the subjective and relational aspects of mental health, and not just the reduction of clinical symptoms. However, it is observed that most of these studies were conducted with male populations^(10,16-20)^. This highlights the gap identified by systematic reviews regarding the need to develop and assess interventions that address the gender-specific needs of women deprived of their liberty^(11)^.

In this regard, the present study seeks to contribute in an innovative manner by proposing a psychosocial intervention aimed exclusively at women deprived of their liberty, based on welcoming and reflective care practices that can be implemented by nurses. It is expected that this intervention will promote self-perception expansion, internal resource strengthening for coping with stressful situations, and mood improvement, considering the conditions of vulnerability and distress imposed by the prison system.

## OBJECTIVES

To analyze the therapeutic potential of a psychosocial intervention protocol developed and administered by nurses.

## METHODS

### Ethical aspects

The study was conducted in accordance with national and international ethical guidelines. It was approved by the Research Ethics Committee of the *Escola de Enfermagem de Ribeirão Preto, Universidade de São Paulo*, under Opinion 5,601,720, issued on August 24, 2022.

### Study design, period, and location

This is a pilot, quantitative, preand post-test study based on the STrengthening the Reporting of OBservational studies in Epidemiology checklist. The study was conducted in a women’s prison complex in a municipality in Minas Gerais. This complex is a prison unit with a capacity for 426 women. As of September 2022, a total of 303 inmates were deprived of their liberty in this penitentiary, predominantly convicted and divided into open, semi-open, and closed regimes. All women deprived of liberty admitted to this penitentiary undergo an initial nursing assessment, aimed at welcoming them, triaging their needs, referring them to other areas when necessary, or requesting examinations. The health unit within this prison has dual management, comprising staff (nurse, nursing technician, social worker, psychologist, dentist, and dental assistant) linked to both the Department of Justice and Public Security (In Portuguese, *Secretaria de Justiça e Segurança Pública* - SEJUSP) and municipal employees (doctor, nurse, nursing technician, psychologist, social worker, dentist, and dental assistant), as stipulated in the PNAISP^(3,12)^. The healthcare team that makes up the PNAISP is expanded, and most of its professionals have a 20-hour work week, while SEJUSP employees work 40 hours a week. Furthermore, this service is characterized as a Basic Health Unit. Thus, emergency or specialized care is referred to external services and handled by the security team responsible for escorting patients.

### Population or sample; inclusion and exclusion criteria

A convenience sample of 12 women was used. Eligibility criteria were having a diagnosed mental disorder, having preserved basic cognitive functions (memory, orientation in time and space), being an adult (18 to 65 years old), and being literate. For the definition of participants in this stage, all women from the aforementioned unit who had a formal diagnosis of some mental disorder were considered as the population (N=104). During the period planned for data collection, the institution considered 12 women eligible to participate in the study (based on behavior, time dedicated to other activities, number of escort agents, room availability, and the availability of the healthcare professional who administered the intervention). An invitation was made in person, and the researcher explained the study’s objectives and data collection procedures. All accepted the invitation and also met the eligibility criteria. Of these, one did not complete the program (she had behavioral problems in her cell during one of the weeks and could not be released), and two did not complete all the questions on the instruments used, resulting in a final sample of nine women.

### Study protocol

The Self-Reporting Questionnaire (SRQ-20) was applied before and after the intervention^(21)^, aiming to identify current symptoms of mental distress, and the Brunel Mood Scale (BRUMS)^(22)^, to measure participants’ mood modulation.

The SRQ-20 was developed by the World Health Organization and validated in Brazil. It consists of 20 items corresponding to emotional and physical symptoms associated with psychiatric conditions. The response options are dichotomous, of the type “yes” (1) or “no” (0), and the final score is obtained from the sum of items, with scores greater than or equal to eight considered suspected cases of mental disorder^(21)^.

The BRUMS, validated in Brazil, consists of 24 items based on a 5-point Likert scale (from 0 = not at all to 4 = extremely), which are divided into negative mood, composed of five factors such as confusion, depression, fatigue, anger, and tension, and positive mood, composed of the vigor factor. The sum of the responses for each factor results in a score that can range from 0 to 16 points^(22)^. Data were collected by the principal researcher, individually, in a private and well-ventilated room, at a time previously agreed upon between the institution and the participant.

The intervention protocol was developed by a nurse with experience in mental health and a doctoral student in psychiatric nursing^(23)^. The theoretical basis of the approaches were cognitive-behavioral techniques (CBTs)^(24-26)^, as well as the propositions and advice derived from psychiatric nursing theories^(27,28)^. The intervention consists of three modules and a workbook. The topics covered are 1) identification of feelings, 2) coexistence with people, and 3) subjectivity. The program’s objective was to facilitate reflection on three pre-defined themes, aiming at better integration between the target population’s emotional and behavioral aspects, as well as promoting self-knowledge and strengthening and/or increasing internal resources for coping with daily adversities, in addition to identifying potential in order to envision future perspectives.

The protocol, in its original version, was configured for application via telephone. However, in this project, it was applied in person through three individual meetings of approximately 20 minutes each. These took place in the nursing care room with the doors closed and the participant seated and without handcuffs. The procedure was observed at all times by the correctional officer who was outside the room, since the door had a glass panel at the top. In these meetings, the topic was explained. Then, participants were invited to reflect on it by completing an activity from the exercise booklet. In the subsequent meeting, participants were invited to talk about the exercise, and the researcher provided at least one positive feedback and advice for behavioral change. Short-duration individual approaches were chosen because this is one of the factors related to adherence, according to previous studies^(29,30)^.

The workbook (a set of tasks for individuals to practice at home what they are learning in the sections and prerogative of the activities that utilize CBT)^(24)^ was named “*Passatempo sentiMental*”, and it includes six activities, such as word searches and crossword puzzles, that address the three themes described above, alternating between technical and playful elements. The material is currently being registered at the Brazilian National Library.

On the first day of the intervention, participants received a printed workbook, and with authorization from the prison management, they were allowed to take it to their cells and keep it with them, as well as fill it out. The workbook was completed using a pen, as authorized by the Minas Gerais Prison System Regulations and Procedures. The workbook was not used for assessment, but as a tool for participants to reflect on and elaborate on the topics covered, considering the principles of CBT^(24)^. In each session, the nurse who implemented the intervention used reflections on the completion of the exercises to provide feedback on that topic, revisiting the concepts presented in the previous session and clarifying doubts to better understand the topic by the participant. At the end, the positive points observed were women’s demonstration of interest and active participation, who requested the continuation of the sessions/intervention and indicated names of cellmates to participate, in addition to strengthening of the professional-patient bond.

### Outcome

Data were double-entered into a Microsoft Excel^®^ spreadsheet, transposed, and analyzed using IBM SPSS Statistics software. The Shapiro-Wilk test results suggested the normality of the distribution of most continuous variables (p-values, respectively, in preand post-test measures: negative mood scores p=0.714 and 0.324; positive mood 0.414 and 0.348; common mental disorder 0.337 and 0.329; number of somatic symptoms 0.108 and 0.074; anxiety 0.012 and 0.028; action/cognition 0.112 and 0.479; and emotion/volition 0.077 and 0.319). Despite this, the boxplot and Q-Q plots were not clearly indicative of a normal distribution, and several authors have pointed out the issue of the low power of normality tests when dealing with small samples (n<30), which is why they recommend the use of non-parametric tests in such samples^(31,32)^. Therefore, to test the difference between preand post-test measures related to symptoms of common mental disorder and mood modulation, the methodological option chosen was the use of the Wilcoxon signed-rank test. The significance level adopted was p < 0.05, with a 95% Confidence Interval (CI).

To analyze clinical and sociodemographic data, descriptive statistics and measures of dispersion (percentages, median, and interquartile range) were used. Regarding the symptoms of common mental disorders, although the SRQ-20 provides a single score that is not divided into dimensions, it is understood that a detailed breakdown of the screened symptoms allows for a more accurate clinical interpretation, especially in the case of this study, which concerns data from nine women. Thus, for the presentation of these results, the questions were grouped into categories considering their content, namely somatic symptoms (questions 1, 2, 3, 7, 18, 19, and 20), anxiety symptoms (questions 4, 5, and 6), symptoms related to cognitive aspects/actions (questions 8, 11, 12, 13, 14, and 16), and symptoms related to emotional issues/volition (questions 9, 10, 15, and 17). The number of symptoms reported by women in each of these categories was plotted on a bar graph with CIs, providing a visual parameter of their variation in preand post-test measurements. To calculate the CI, due to the sample size (n<30) and the fact that the population standard deviation was unknown, Student’s t-distribution was used^(33)^, Considering eight degrees of freedom (n-1), 95% confidence, corresponding critical value (two-sided) in the t-distribution table (2.306). The standard error (SE) was calculated using the formula “SE=s/√n” (s is the sample standard deviation, and n, the sample size). The CI was obtained from the formula “CI = mean ± (critical value * standard error).

## RESULTS

### Participant characteristics

**Figure 1:**
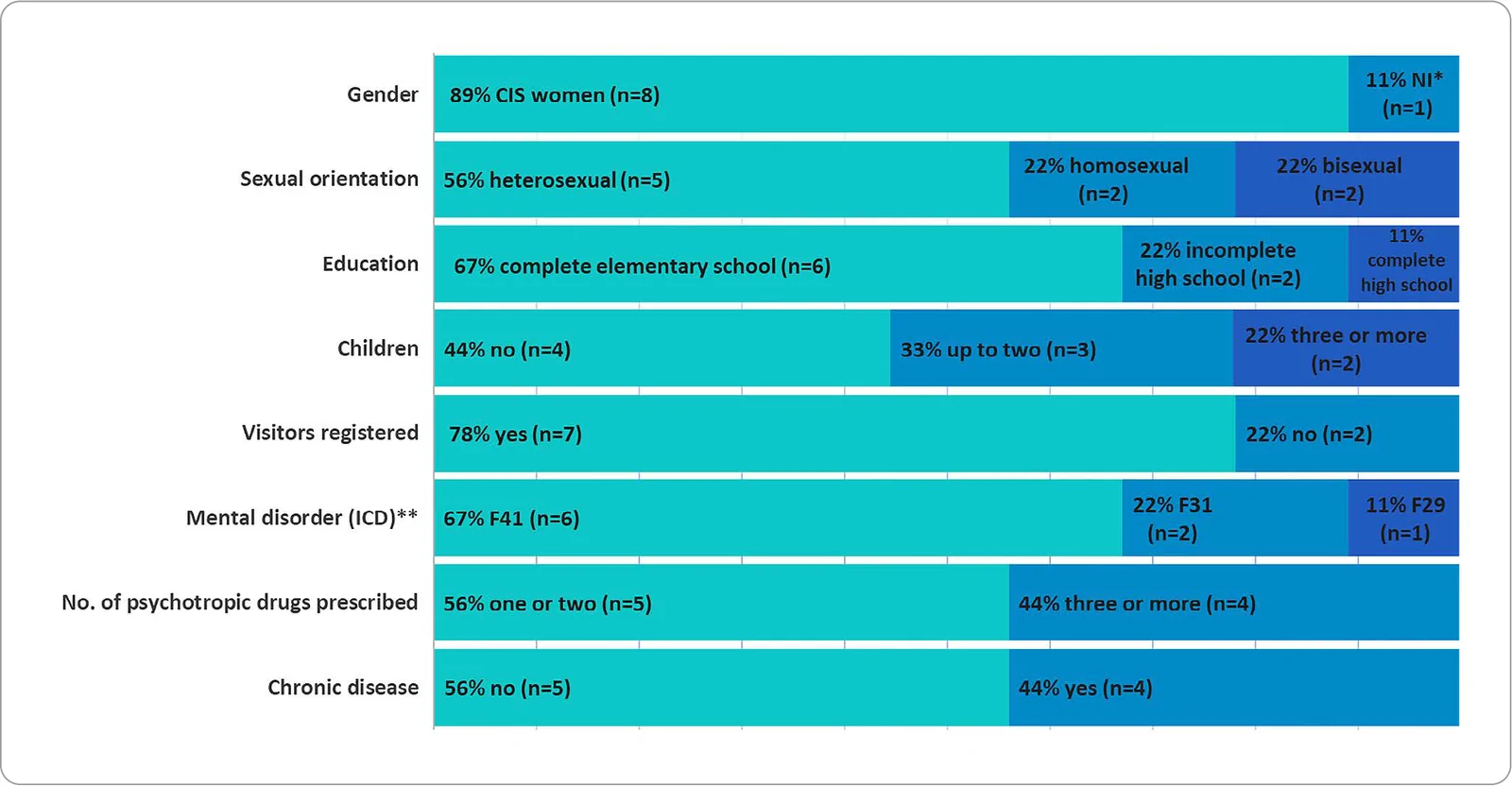
Distribution of participants according to sociodemographic characteristics, Belo Horizonte, Minas Gerais, Brazil, 2023

Concerning age, the median age was 27 years (interquartile range 24 to 42.5 years). The median length of incarceration was 10.77 years (interquartile range 8.24 to 20.70). All were single, self-identified as black or brown, had a psychiatric diagnosis, and were undergoing treatment with psychotropic drugs. Figure 1 presents their other sociodemographic characteristics.

*NI - not informed; ** F41 - other anxiety disorders; F31 - bipolar affective disorder; F29 - unspecified non-organic psychosis.

### Preand post-test measurements

In relation to mood, in descriptive terms, improvement was identified in all domains analyzed, with particular emphasis on domains 2 (depression) and 5 (tension), which showed a statistically significant reduction in follow-up. The overall score related to negative mood also showed a statistically significant decrease compared to the baseline (Table 1).

**Table 1:** Median and interquartile range of preand post-test measurements, Belo Horizonte, Minas Gerais, Brazil, 2023

Variables	Median (interquartile range)		Z	*p* value
	Baseline	Follow-up		
SRQ-20 score	10.00 (8.00-13.00)	10.00 (6.50-10.50)	-0.70	0.48
Negative mood	44.00 (20.50-50.50)	24.00 (12.50-42.00)	-2.31	0.02
Confusion	8.00 (3.00-11.00)	4.00 (3.00-10.00)	-1.61	0.11
Depression	10.00 (5.00-13.50)	3.00 (2.00-12.00)	-2.20	0.03
Fatigue	7.00 (1.00-13.00)	3.00 (1.50-5.50)	-1.27	0.21
Anger	5.00 (2.00-8.50)	3.00 (1.00-6.50)	-1.70	0.09
Tension	9.00 (6.50-13.00)	7.00 (4.00-9.00)	-2.03	0.04
Positive mood	9.00 (3.50-15.00)	8.00 (6.00-15.00)	-1.62	0.11
Vigor	9.00 (3.50-15.00)	8.00 (6.00-15.00)	-1.62	0.11

*SRQ-20 - Self-Reporting Questionnaire.*

**Figure 2:**
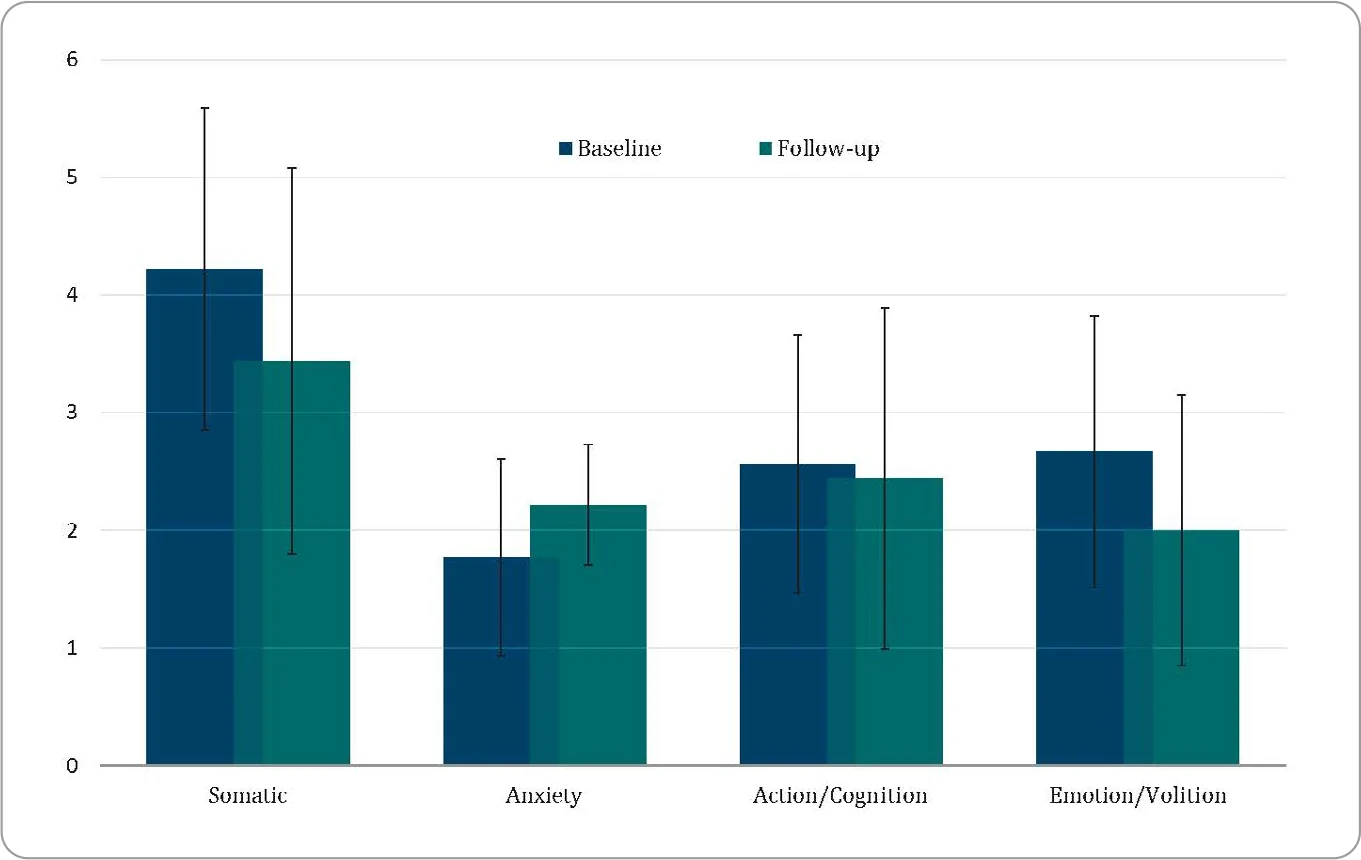
Mean number of symptoms of common mental disorders and their respective confidence intervals at baseline and follow-up, considering the categories proposed in the analysis, Belo Horizonte, Minas Gerais, Brazil, 2023

As for symptoms of common mental disorders, in descriptive terms, a decrease was observed in those related to somatic, behavioral, and emotional aspects, and an increase in those related to anxiety (Figure 2), in addition to a reduction in overall SRQ-20 scores in the post-test measure. This reduction, although clinically relevant, did not show statistical significance (Table 1).

## DISCUSSION

The sample for this study consisted of young women of reproductive age, with low levels of education, of African descent, and mothers, supporting previous research regarding the influence of race/color on police assessment^(34)^ regarding this subgroup reflecting the characteristics of the majority of women in our country, which is largely marked by social inequality^(35,36)^.

In relation to health, the presence of chronic diseases concomitant with mental disorders, especially anxiety and mood disorders among incarcerated women, supports findings from national studies^(4,5)^ and international^(9-11)^. These studies highlight the need to improve access to and quality of healthcare in prison settings, especially among groups most vulnerable to deprivation of liberty, such as women, mothers, black people, and LGBTQIA+ individuals^(4-6,9-11,37,38)^.

Research indicates particularly negative consequences for mothers’ mental health due to the maternal separation imposed by the prison system, which leads to feelings of loss, sadness, and abandonment^(37,38)^. These women also report receiving fewer family visits^(6)^, and face significant limitations in accessing healthcare, including insufficient prenatal care and a lack of guidance focused on health promotion^(4)^.

In the case of the LGBTQIA+ population, a recent review has shown that the prison experience is marked by invisibility and multiple forms of violation of fundamental rights. Among the reported forms of violence are disrespect for the use of chosen names, the imposition of specific haircuts, lack of access to hormone treatments, the lack of specific cells or wings, and the scarcity of official information about this group within the prison system. These factors intensify psychological distress and constitute a double punishment^(39)^.

Additionally, 64% of people in the Brazilian prison system are black, and this overrepresentation reflects the persistent effects of structural racism that permeates Brazilian society. The black population is historically more exposed to poverty, social inequality, state violence, and exclusion, which contributes to higher rates of incarceration and poorer health conditions^(34,40)^.

Given this scenario, it is noteworthy that the intersection of gender, motherhood, race, and sexuality exacerbates vulnerability and the risk of mental illness in prisons. Therefore, it is essential that psychosocial policies and interventions focused on the mental health of the population deprived of liberty consider these dimensions in an integrated and sensitive manner, taking into account the specific needs of these groups.

The fact that depression and tension emerged as the most prominent domains in terms of participants’ mood certainly reflects issues related to precarious infrastructure and relationships, as well as the hostile and violent characteristics of most prison environments^(3,6,7,37,38)^. These aspects contribute to worsening psychological distress.

In this context, the significant reduction in depression and tension scores after the intervention highlights the relevance of the proposed approach, especially considering the sample profile and the context of deprivation of liberty. This positive effect can be attributed to the intervention structure, based on the principles of CBT and psychiatric nursing, which sought to foster moments of reflection on the identification and expression of feelings, the development of social skills, and the subjective processing of experiences. Thus, the reductions observed in these domains suggest that participants expanded their capacity for emotional perception and affective self-regulation, corroborating studies that employed similar interventions^(16-19)^.

In contrast, the other domains (confusion, fatigue, anger, and vigor) proved more resistant to change. This stability may be related both to the fact that these dimensions are often more influenced by physical and environmental aspects, and to the structural nature of prison stressors, such as rigid routines, lack of meaningful stimuli, deprivation of liberty, and lack of perspective regarding the future^(3,6,7,37,38)^. These factors can limit the reach of short-term interventions focused on subjective aspects.

Regarding the lack of a significant reduction in symptoms of mental disorders, it is possible that the duration of exposure to the intervention, the sample size, and the complexity of the participants’ psychological conditions limited the magnitude of the effect. However, the changes observed in descriptive terms reinforce the therapeutic potential of the protocol, indicating sensitivity of the somatic, behavioral, and emotional dimensions to the proposed actions.

International studies converge on this point, demonstrating that approaches based on CBT promote benefits related to active listening, strengthening the internal locus of control, and reframing personal experiences^(16,18,41,42)^. Other forms of intervention, such as drama therapy^(10)^ and horticultural therapy^(17)^, they have also shown effectiveness in promoting emotional expression, restructuring narratives, and developing insights and cognitive skills^(10,17,19)^. It is worth adding that the review study also highlighted the importance and potential of approaches that encourage reflection on goals and life purpose^(11)^; this aspect is also inherent to the intervention proposed in this study.

For future improvements, the incorporation of co-produced methodologies is recommended^(20)^, in addition to approaches based on strengths^(17)^ and strategies based on the logic of peer support^(41,42)^, which can increase participant engagement and enhance the therapeutic effects of actions aimed at promoting mental health in the prison context.

For the implementation and sustainability of such interventions, the central role of nursing, especially psychiatric nursing, stands out, as its practice is historically grounded in interpersonal relationships, therapeutic communication, and empathetic support. Furthermore, care is guided by a holistic and relational perspective, in which professionals work alongside individuals to foster the development of coping strategies and the strengthening of autonomy. This process involves considering the unique characteristics of each individual, their living conditions, and the resources available in different social and economic contexts, continuously collaborating with other members of the multidisciplinary team^(14)^.

Recent studies reinforce the leading role of nurses in coordinating care between healthcare services and the prison system^(13)^, as well as in conducting interventions aimed at promoting mental health and reducing stigma associated with psychological distress in contexts of deprivation of liberty^(15)^. Hence, the innovative nature of this intervention lies, in part, in the fact that it was implemented by a nurse, demonstrating psychiatric nursing’s capacity to conduct structured and effective therapeutic practices capable of promoting spaces for listening, reflection, and reinterpretation of the experience of deprivation of liberty.

### Study limitations

The limitations of this study are its pilot design, consisting of preand post-tests without a control group, which involved a small sample size and allowed only non-parametric analyses, making it impossible to infer causality among the observed aspects. Furthermore, the restriction regarding the inclusion of other participants, who might even have behavioral problems, limited the evaluative possibilities of the intervention. Additionally, improvements shown by participants may be related to other factors in the prison context that were not considered in this study. Despite these limitations, the clinical relevance of the findings of this study is noteworthy. Future research is suggested to assess the results of this intervention in specific diagnostic groups, with larger samples, better effect sizes, and more robust designs, in order to provide more precise parameters regarding its therapeutic potential.

### Contributions to nursing

The study findings offer several relevant contributions to nursing practice, especially in the context of mental healthcare in the female prison system. Firstly, the potential of brief interventions conducted by nurses was highlighted, since, even with the study limitations, a significant reduction in tension and depression scores was observed, suggesting that approaches based on CBT and psychiatric nursing counseling may be viable and effective in this context. Furthermore, the tasks proposed during the intervention, using the “*Passatempo sentiMental*”, demonstrated potential for autonomous continuity, allowing participants to reflect on their experiences and emotions outside of the sessions, promoting inmates’ leading role in their care process.

## CONCLUSIONS

As for the intervention carried out, although it is a study protocol and the results are related only to the application of this pilot study, it was possible to observe improvement in all the measures assessed, with statistical significance in the depression and tension items of the BRUMS. A decrease was also observed in the somatic, behavioral, and emotional aspects of the symptoms of common mental disorders, suggesting the potential of the proposed intervention as a non-pharmacological therapeutic option for nurses working in the prison system.

## Data Availability

The research data are available only upon request.
