# Supplementary material for: Therapeutic potential of a psychosocial intervention in the mental health of women deprived of liberty
Source: Rev Bras Enferm. 2026 Sep 18;79:e20250348. doi: 10.1590/0034-7167-2025-0348 (PMC13588629; doi:10.1590/0034-7167-2025-0348)
Supplement: Translation

# Translation: Potencial terapêutico de uma intervenção psicossocial na saúde mental de mulheres privadas de liberdade

**DOI:** 10.1590/0034-7167-2025-0348pt

**Authors:** Maria Theresa Veloso Figueiredo de Carvalho, Jaqueline Lemos de Oliveira, Janaína Cristina Pasquini de Almeida, Antonio José Correa de Pauli, Bianca Moretti Tomiazzi, Jacqueline de Souza

**Affiliations:** I Universidade de São Paulo. Ribeirão Preto, São Paulo, Brasil; II Universidade de São Paulo. São Paulo, São Paulo, Brasil

**Keywords:** Intervenção Psicossocial, Saúde Mental, Mulheres, Prisioneiros, Enfermagem.

## Abstract

**Objetivos:**

analisar o potencial terapêutico de um protocolo de intervenção psicossocial desenvolvido e administrado por enfermeiras.

**Métodos:**

estudo piloto, quantitativo, do tipo prée pós-teste, aprovado pelo Comitê de Ética e realizado com 12 mulheres privadas de liberdade em um Complexo Penitenciário Feminino em um município de Minas Gerais. Foram coletados dados sociodemográficos e referentes aos sintomas de transtorno mental comum e modulação do humor. Utilizaram-se estatística descritiva, medidas de dispersão e teste não paramétrico para a análise de dados.

**Resultados:**

nove mulheres completaram o programa proposto. Identificou-se melhoria em todos os domínios analisados em relação à modulação do humor, com destaque para os domínios depressão e tensão. Quanto aos sintomas de transtorno mental comum, houve redução em termos descritivos, porém sem significância estatística.

**Conclusões:**

os resultados sugerem que a intervenção proposta apresenta potencial para ser considerada como uma opção terapêutica não farmacológica pelos enfermeiros que atuam no sistema prisional.

## INTRODUÇÃO

No Brasil, a quantidade de pessoas privadas de liberdade equivale a 500.381, das quais 31.181 são mulheres^(1)^. Apenas 7% das unidades prisionais brasileiras são exclusivamente destinadas para mulheres^(2)^, embora a Lei de Execução Penal (Lei n.º 7.210, de 11 de julho de 1984) e a Política Nacional de Atenção às Mulheres em Situação de Privação de Liberdade e Egressas do Sistema Prisional prevejam unidades prisionais específicas para o público feminino, considerando as situações de exclusão e violação de direitos frequentemente vivenciadas por mulheres em unidades prisionais mistas^(3-6)^.

A disparidade encontrada no tratamento oferecido a mulheres privadas de liberdade reproduz as desigualdades vivenciadas na sociedade em geral e escancara o viés masculino que permeia as regras aplicadas a essas mulheres, ignorando as particularidades que as interseccionam como classe, cultura e gênero, o que resulta na marginalização e subalternização das mesmas^(7)^. Vários pesquisadores têm se dedicado especificamente ao público feminino privado de liberdade. Eles ressaltam a necessidade de as equipes de saúde priorizarem esse público, considerando o histórico de traumas, abusos e negligências geralmente sofridos ao longo da vida, associados ao ambiente prisional hostil e à violação dos direitos humanos, como a superlotação e a dificuldade de acesso à assistência e ao tratamento de saúde, bem como a itens básicos de higiene^(3-6)^.

Estudos demonstram que tais fatores de vulnerabilidade contribuem para a intensificação ou o desenvolvimento de transtornos mentais, e alguns casos culminam em atitudes autodestrutivas^(4-6,8-11)^. Pesquisa realizada com mulheres privadas de liberdade no Ceará identificou a prevalência de 68,4% de transtornos mentais comuns^(4)^, enquanto estudo conduzido em uma penitenciária do Pará revelou que 50% apresentavam sintomas indicativos de ansiedade, depressão e/ou estresse^(5)^. Em âmbito internacional, investigações realizadas em penitenciárias da Noruega e do Reino Unido apontaram prevalências de transtornos psiquiátricos de 56% e 76%, respectivamente^(9,10)^. Ademais, revisão sistemática indicou o transtorno de estresse pós-traumático como o problema de saúde mental mais frequente entre mulheres privadas de liberdade^(11)^. Tais achados, aliados ao estigma social e à descontinuidade do cuidado em saúde mental, reforçam a necessidade de que a saúde mental seja tratada como prioridade nas políticas e práticas de atenção à saúde prisional, com enfoque específico nas demandas das mulheres privadas de liberdade.

Contudo, o acesso à saúde no âmbito prisional carece de ser acessível e provido em condições aproximadas ao que é ofertado para a população em geral mesmo após a implementação da Política Nacional de Atenção Integral à Saúde das Pessoas Privadas de Liberdade no Sistema Prisional (PNAISP)^(12)^ em 2014, que objetiva a garantia do direito à saúde para todas as pessoas reclusas no sistema prisional, por meio do acesso ao Sistema Único de Saúde. Entre os profissionais que compõem a equipe mínima estabelecida pela PNAISP, destaca-se o enfermeiro como um profissional de extrema importância para a assistência em saúde mental prisional, uma vez que, ao pensar o cuidado integral e humanizado, possibilita uma assistência coerente com as reais necessidades de saúde dessa população, minimizando os efeitos negligentes e proporcionando o bem-estar, o resgate da condição digna de vida e a promoção de saúde mental, conforme apontado em estudos prévios^(13-15)^.

Em relação às intervenções de saúde mental para o público privado de liberdade, estudos que utilizaram estratégias baseadas na Teoria Cognitivo-Comportamental^(16)^, dramaterapia^(10)^, hortiterapia^(17)^ e acolhimento^(18)^ identificaram resultados positivos para a melhoria do autocontrole, da regulação emocional, da autoestima, da satisfação com a vida, do bem-estar subjetivo e do desenvolvimento de habilidades sociais^(10,16-18)^. Tais achados evidenciam a importância de considerar os aspectos subjetivos e relacionais da saúde mental, e não apenas a redução dos sintomas clínicos. Entretanto, observa-se que a maioria dessas pesquisas foi conduzida com o público masculino^(10,16-20)^, o que evidencia a lacuna apontada por revisão sistemática sobre a necessidade de desenvolver e avaliar intervenções que abordem as especificidades de gênero das mulheres em privação de liberdade^(11)^.

Nesse sentido, o presente estudo busca contribuir de forma inovadora, ao propor uma intervenção psicossocial voltada exclusivamente a mulheres privadas de liberdade, fundamentada em práticas de cuidado acolhedoras e reflexivas, possíveis de serem implementadas por enfermeiros. Espera-se que essa intervenção favoreça a ampliação da percepção de si, o fortalecimento de recursos internos para o enfrentamento de situações estressoras e a melhoria do estado de humor, considerando as condições de vulnerabilidade e de sofrimento impostas pelo sistema prisional.

## OBJETIVOS

Analisar o potencial terapêutico de um protocolo de intervenção psicossocial desenvolvido e administrado por enfermeiras.

## MÉTODOS

### Aspectos éticos

O estudo foi conduzido conforme diretrizes éticas nacionais e internacionais. Foi aprovado pelo Comitê de Ética em Pesquisa da Escola de Enfermagem de Ribeirão Preto da Universidade de São Paulo, sob Parecer nº 5.601.720, emitido em 24 de agosto de 2022.

### Desenho, período e local do estudo

Trata-se de estudo piloto, quantitativo, do tipo prée pós-teste, baseado na lista de verificação *STrengthening the Reporting of OBservational studies in Epidemiology*. O estudo foi desenvolvido em um Complexo Penitenciário Feminino de um município de Minas Gerais. Tal complexo é uma unidade prisional com capacidade para 426 mulheres. Até setembro de 2022, estavam privadas de liberdade nessa penitenciária um total de 303 internas, sendo essas predominantemente condenadas, divididas nos regimes aberto, semiaberto e fechado. Todas as mulheres privadas de liberdade admitidas nessa penitenciária passam por um primeiro atendimento de enfermagem, com vistas ao acolhimento, triagem das demandas, encaminhamento para as demais áreas, quando necessário, ou solicitação de exames. A unidade de saúde dessa unidade prisional possui gestão dupla, sendo composta por servidores (enfermeiro, técnico de enfermagem, assistente social, psicólogo, dentista e auxiliar em saúde bucal) vinculados tanto à Secretaria de Justiça e Segurança Pública (SEJUSP) quanto a funcionários do município (médico, enfermeiro, técnico de enfermagem, psicólogo, assistente social, dentista e auxiliar em saúde bucal), como previsto na PNAISP^(3,12)^. A equipe de saúde que compõe o PNAISP é ampliada, e a maioria de seus profissionais possui carga horária de 20 horas, enquanto os servidores da SEJUSP realizam 40 horas semanais. Além disso, esse serviço se caracteriza como Unidade Básica de Saúde. Assim, atendimentos de urgência/emergência ou especializados são referenciados para serviços externos e conduzidos pela equipe de segurança responsável pela escolta.

### População ou amostra; critérios de inclusão e exclusão

Foi adotada uma amostra de conveniência composta por 12 mulheres. Os critérios de elegibilidade foram ter um transtorno mental diagnosticado, estar com as funções cognitivas básicas preservadas (memória, orientação no tempo e no espaço), ser adulta (18 a 65 anos) e alfabetizada. Para a definição das participantes dessa etapa, foram consideradas como população todas as mulheres da referida unidade que tinham o diagnóstico formal de algum transtorno mental (N=104). No período previsto para a coleta de dados, a instituição considerou como aptas para a participação do estudo (comportamento, tempo dedicado para outras atividades, contingente de agentes para escolta, disponibilidade de sala e da profissional da equipe de saúde que aplicou a intervenção) 12 mulheres. O convite foi realizado pessoalmente, e a pesquisadora explicou os objetivos do estudo e os procedimentos de coleta de dados. Todas aceitaram o convite e também atendiam aos critérios de elegibilidade. Destas, uma não completou o programa (teve problemas de comportamento na cela em uma das semanas e não pôde ser liberada) e duas não preencheram todas as questões dos instrumentos utilizados, perfazendo uma amostra final de nove mulheres.

### Protocolo do estudo

Nos momentos prée pós-intervenção, foram aplicados o *Self-Reporting Questionnaire* (SRQ-20)^(21)^, visando identificar os sintomas de sofrimento mental vigentes, e a *Brunel Mood Scale* (BRUMS)^(22)^, para mensurar a modulação humor das participantes.

O SRQ-20 foi desenvolvido pela Organização Mundial da Saúde e validado no Brasil. É composto por 20 itens correspondentes a sintomas emocionais e físicos associados a quadros psiquiátricos. As opções de resposta são dicotômicas, do tipo “sim” (1) ou “não” (0), e o escore final é obtido a partir da soma dos itens, sendo considerados casos suspeitos de transtorno mental os escores maiores ou iguais a oito^(21)^.

A BRUMS, validada no Brasil, é composta por 24 itens baseados em uma escala Likert de 5 pontos (de 0 = nada a 4 = extremamente), que são divididos em humor negativo, composto por cinco fatores, como confusão, depressão, fadiga, raiva e tensão, e humor positivo, composto pelo fator vigor. A soma das respostas de cada fator resulta em um escore que pode variar de 0 a 16 pontos^(22)^. Os dados foram coletados pela pesquisadora principal, individualmente, em uma sala privativa e arejada, em horário previamente acordado entre a instituição e a participante.

O protocolo da intervenção foi desenvolvido por uma enfermeira com experiência na área de saúde mental e uma doutoranda em enfermagem psiquiátrica^(23)^. O embasamento teórico das abordagens foram as técnicas cognitivas-comportamentais (TCCs)^(24-26)^, bem como as proposições e aconselhamentos oriundos das teorias de enfermagem psiquiátrica^(27,28)^. A intervenção é composta por três módulos e um caderno de exercícios. Os temas abordados são 1) identificação dos sentimentos, 2) convivência com as pessoas e 3) subjetividade. O objetivo do programa foi viabilizar reflexões sobre três temas previamente definidos, visando à melhor integração entre os aspectos emocionais e comportamentais da população-alvo, bem como à promoção do autoconhecimento e ao fortalecimento e/ou aumento de recursos internos próprios para o enfrentamento das adversidades cotidianas, além da identificação de potencialidades, a fim de vislumbrar perspectivas futuras.

O protocolo, em sua versão original, foi configurado para aplicação via telefone. No entanto, no presente projeto, foi aplicado na modalidade presencial por meio de três encontros individuais de, aproximadamente, 20 minutos. Esses ocorreram na sala de atendimento da enfermagem de portas fechadas e com a participante sentada e sem algemas. O procedimento foi visualizado a todo momento pelo policial penal que se encontrava ao lado de fora da sala, visto que a porta possuía um vidro na parte superior. Em tais encontros, foi realizada a explanação do tema. Em seguida, a participante era convidada a refletir sobre o mesmo a partir da realização de uma atividade do caderno de exercícios. No encontro posterior, a participante era convidada a falar sobre o exercício, e a pesquisadora proporcionava ao menos um *feedback* positivo e um aconselhamento para mudança de comportamento. Optaram-se por abordagens individuais de curta duração, por ser um dos fatores relacionados à adesão, de acordo com estudos prévios^(29,30)^.

O caderno de exercícios (conjunto de tarefas para o indivíduo praticar em casa o que está aprendendo nas seções e prerrogativa das atividades que utilizam TCC)^(24)^ foi nomeado como “Passatempo sentiMental”, e conta com seis atividades, como caça-palavras e palavras cruzadas, que abordam as três temáticas anteriormente descritas, intercalando o técnico e o lúdico. O material está em fase de registro na Biblioteca Nacional.

As participantes receberam no primeiro dia da intervenção o caderno de exercícios impresso, e com autorização da direção da unidade prisional, puderam levar para suas celas e guardar consigo, bem como preencher o material. O preenchimento era realizado através do uso de caneta, conforme autorização emitida pelo Regulamento e Normas de Procedimentos do Sistema Prisional de Minas Gerais. O caderno de exercícios não foi utilizado para avaliação, mas como ferramenta para que a participante refletisse e elaborasse os temas abordados considerando os preceitos da TCC^(24)^. Em cada encontro, a enfermeira que aplicou a intervenção utilizava as reflexões sobre a realização dos exercícios para basear o *feedback* daquele tema, retomando os conceitos apresentados no encontro anterior e elucidando dúvidas para melhor elaboração do tema pela participante. Ao término, os pontos positivos observados foram a demonstração de interesse e a participação ativa das mulheres, que pediram pela continuidade dos encontros/intervenção e indicaram nomes de colegas de cela para participar, além do fortalecimento do vínculo profissional-paciente.

### Desfecho

Os dados foram duplamente digitados em uma planilha do *Microsoft Excel^®^*, transpostos e analisados utilizando o *software* IBM SPSS *Statistics*. Os resultados do teste de Shapiro-Wilk sugeriram a normalidade da distribuição da maioria das variáveis contínuas (valores de p, respectivamente, nas medidas de prée pós-teste: escores de humor negativo p=0,714 e 0,324; humor positivo 0,414 e 0,348; transtorno mental comum 0,337 e 0,329; número de sintomas somáticos 0,108 e 0,074; ansiedade 0,012 e 0,028; ação/cognição 0,112 e 0,479; e emoção/volição 0,077 e 0,319). Apesar disso, os gráficos *boxplot* e Q-Q *plots* não foram claramente indicativos de distribuição normal, e vários autores têm apontado a questão do baixo poder dos testes de normalidade quando se tratam de amostras pequenas (n<30), razão pela qual recomendam a utilização de testes não paramétricos em tais amostras^(31,32)^. Desse modo, para testar a diferença entre as medidas de prée pós-teste relacionadas aos sintomas de transtorno mental comum e modulação do humor, a opção metodológica foi pela utilização do *Wilcoxon signed-rank test.* O nível de significância adotado foi de p < 0,05, com Intervalo de Confiança (IC) de 95%.

Para analisar os dados clínicos e sociodemográficos, utilizaram-se estatísticas descritivas e medidas de dispersão (percentuais, mediana e intervalo interquartil). Quanto aos sintomas de transtorno mental comum, embora a SRQ-20 proporcione um escore único que não se divide em dimensões, entende-se que um detalhamento dos sintomas rastreados possibilita uma leitura clínica mais apurada, sobretudo no caso do presente estudo, que diz respeito aos dados de nove mulheres. Assim, para a apresentação de tais resultados, optou-se pelo agrupamento das questões em categorias considerando o teor das mesmas, a saber sintomas somáticos (questões 1, 2, 3, 7, 18, 19 e 20), sintomas de ansiedade (questões 4, 5 e 6), sintomas relacionados aos aspectos cognitivos/ações (questões 8, 11, 12, 13, 14 e 16) e sintomas relacionados às questões emocionais/volição (questões 9, 10, 15 e 17). O número de sintomas referidos pelas mulheres em cada uma dessas categorias foi plotado em um gráfico de barras com ICs, proporcionando um parâmetro visual de sua variação nas medidas do prée pós-testes. Para calcular o IC, devido ao tamanho amostral (n<30) e que o desvio padrão da população não era conhecido, foi utilizada a distribuição t de Student^(33)^, considerando oito graus de liberdade (n-1), 95% de confiança, valor crítico correspondente (*two-sided*) na tabela de distribuição t (2,306). O erro padrão (SE) foi calculado utilizando a fórmula “SE=s/√n” (s é o desvio padrão da amostra, e n, o tamanho amostral). o IC foi obtido a partir da fórmula “IC = média ± (valor crítico * erro padrão).

## RESULTADOS

### Características das participantes

Quanto à idade, a mediana da distribuição foi de 27 anos (intervalo interquartil de 24 a 42,5 anos). O tempo de reclusão teve mediana de 10,77 anos (intervalo interquartil de 8,24 a 20,70). Todas eram solteiras, se autodeclaravam pretas ou pardas, tinham diagnóstico psiquiátrico e faziam tratamento com psicotrópicos. A Figura 1 apresenta as demais características sociodemográficas das mesmas.

**Figura 1:** Distribuição das participantes de acordo com as características sociodemográficas, Belo Horizonte, Minas Gerais, Brasil, 2023

*NI - não informado; ** F41 - outros transtornos ansiosos; F31 - transtorno afetivo bipolar; F29 - psicose não orgânica não especificada.

### Medidas prée pós-teste

Quanto ao humor, em termos descritivos, identificou-se melhoria em todos os domínios analisados, com destaque para os domínios 2 (depressão) e 5 (tensão), que apresentaram redução estatisticamente significante no *follow-up*. O escore geral relacionado ao humor negativo também teve decréscimo estatisticamente significante em relação à linha de base (Tabela 1).

**Tabela 1:** Mediana e intervalo interquartil das medidas prée pós-teste, Belo Horizonte, Minas Gerais, Brasil, 2023

Variáveis	Mediana (intervalo interquartil)		Z	Valor de *p*
	Linha de base	*Follow-up*		
Escore do SRQ-20	10,00 (8,00-13,00)	10,00 (6,50-10,50)	-0,70	0,48
Humor negativo	44,00 (20,50-50,50)	24,00 (12,50-42,00)	-2,31	0,02
Confusão	8,00 (3,00-11,00)	4,00 (3,00-10,00)	-1,61	0,11
Depressão	10,00 (5,00-13,50)	3,00 (2,00-12,00)	-2,20	0,03
Fadiga	7,00 (1,00-13,00)	3,00 (1,50-5,50)	-1,27	0,21
Raiva	5,00 (2,00-8,50)	3,00 (1,00-6,50)	-1,70	0,09
Tensão	9,00 (6,50-13,00)	7,00 (4,00-9,00)	-2,03	0,04
Humor positivo	9,00 (3,50-15,00)	8,00 (6,00-15,00)	-1,62	0,11
Vigor	9,00 (3,50-15,00)	8,00 (6,00-15,00)	-1,62	0,11

*SRQ-20 - Self-Reporting Questionnaire.*

Quanto aos sintomas de transtorno mental comum, em termos descritivos, observaram-se decréscimo dos relacionados aos aspectos somáticos, comportamentais e emocionais e aumento dos relacionados à ansiedade (Figura 2), além de redução dos escores gerais do SRQ-20 na medida pós-teste. Essa redução, embora com relevância clínica, não apresentou significância estatística (Tabela 1).

**Figura 2:** Número médio de sintomas de transtorno mental comum e respectivos Intervalos de Confiança na linha de base e no *follow-up*, considerando as categorias propostas na análise, Belo Horizonte, Minas Gerais, Brasil, 2023

## DISCUSSÃO

A amostra do presente estudo foi composta por mulheres jovens, em idade produtiva e reprodutiva, com baixa escolaridade, afrodescendentes e mães, corroborando pesquisas prévias tanto no que tange aos resultados da influência dos quesitos raça-cor na avaliação da polícia^(34)^ quanto em relação a esse subgrupo refletir as características da maioria das mulheres do nosso país, amplamente marcado pela desigualdade social^(35,36)^.

Em relação à saúde, a presença de doenças crônicas concomitantes a transtornos mentais, especialmente os transtornos de ansiedade e do humor entre mulheres privadas de liberdade, corrobora achados de estudos nacionais^(4,5)^ e internacionais^(9-11)^. Esses estudos evidenciam a necessidade de aprimorar o acesso e a qualidade do cuidado em saúde nos ambientes prisionais, especialmente entre grupos mais vulneráveis à privação de liberdade, como mulheres, mães, pessoas negras e integrantes da população LGBTQIA+^(4-6,9-11,37,38)^.

Pesquisas apontam consequências particularmente negativas para a saúde mental de mulheres com filhos devido à separação materna imposta pelo sistema prisional, que acarreta sentimento de perda, tristeza e abandono^(37,38)^. Essas mulheres também relatam receber menos visitas familiares^(6)^, e enfrentam limitações importantes no acesso ao cuidado em saúde, incluindo a realização insuficiente de consultas pré-natais e a falta de orientações voltadas à promoção da saúde^(4)^.

No caso da população LGBTQIA+, revisão recente evidenciou que a experiência prisional é marcada por invisibilidade e múltiplas formas de violação de direitos fundamentais. Entre as violências relatadas, destacam-se o desrespeito ao uso do nome social, a imposição de cortes de cabelo, a ausência de acesso a tratamentos hormonais, a falta de celas ou alas específicas e a escassez de informações oficiais sobre esse grupo no sistema prisional. Esses fatores intensificam o sofrimento psíquico e configuram uma dupla punição^(39)^.

Adicionalmente, 64% das pessoas no sistema prisional no Brasil são negras, e essa sobrerrepresentação reflete os efeitos persistentes do racismo estrutural, que atravessa a sociedade brasileira. A população negra é historicamente mais exposta à pobreza, à desigualdade social, à violência do Estado e à exclusão, o que contribui para taxas mais elevadas de privação de liberdade e piores condições de saúde^(34,40)^.

Diante desse cenário, destaca-se que a interseção entre gênero, maternidade, raça e sexualidade potencializa a vulnerabilidade e o risco de adoecimento mental no cárcere. Assim, torna-se fundamental que políticas e intervenções psicossociais voltadas à saúde mental da população privada de liberdade considerem essas dimensões de forma integrada e sensível às especificidades desses grupos.

O fato de a depressão e a tensão terem figurado como os domínios mais proeminentes no que se refere ao humor das participantes certamente reflete as questões relacionadas à precariedade da infraestrutura e das relações, bem como às características de hostilidade e violência da maioria dos ambientes prisionais^(3,6,7,37,38)^. Tais aspectos contribuem para o agravamento do sofrimento psíquico.

Nesse contexto, a redução significativa dos escores de depressão e tensão após a intervenção evidencia a relevância da proposta desenvolvida, sobretudo considerando o perfil da amostra e o contexto de privação de liberdade. Esse efeito positivo pode ser atribuído à estrutura da intervenção, fundamentada nos princípios da TCC e da enfermagem psiquiátrica, que buscou favorecer momentos de reflexão sobre a identificação e a expressão de sentimentos, o desenvolvimento de habilidades sociais e a elaboração subjetiva de experiências. Dessa forma, as reduções observadas nesses domínios sugerem que as participantes ampliaram sua capacidade de percepção emocional e de autorregulação afetiva, corroborando estudos que empregaram intervenções semelhantes^(16-19)^.

Em contrapartida, os demais domínios (confusão, fadiga, raiva e vigor) se mostraram mais resistentes à mudança. Essa estabilidade pode estar relacionada tanto ao fato de que tais dimensões são frequentemente mais influenciadas por aspectos físicos e ambientais quanto ao caráter estrutural dos estressores do cárcere, como a rotina rígida, a ausência de estímulos significativos, a privação de liberdade e a falta de perspectiva quanto ao futuro^(3,6,7,37,38)^. Esses fatores podem limitar o alcance de intervenções de curta duração e centradas em aspectos subjetivos.

Quanto à ausência de redução significativa dos sintomas de transtornos mentais, é possível que o tempo de exposição à intervenção, o tamanho da amostra e a complexidade das condições psíquicas das participantes tenham limitado a magnitude do efeito. No entanto, as mudanças observadas em termos descritivos reforçam o potencial terapêutico do protocolo, indicando sensibilidade das dimensões somáticas, comportamentais e emocionais às ações propostas.

Estudos internacionais convergem nesse sentido ao demonstrarem que abordagens baseadas na TCC promovem benefícios relacionados à escuta qualificada, ao fortalecimento do lócus de controle interno e à ressignificação das experiências pessoais^(16,18,41,42)^. Outras modalidades de intervenção, como a dramaterapia^(10)^ e a hortiterapia^(17)^, também mostraram eficácia ao favorecer a expressão emocional, a reestruturação de narrativas, e o desenvolvimento de *insights* e habilidades cognitivas^(10,17,19)^. Vale acrescentar que estudo de revisão também apontou a importância e potencial de abordagens que proporcionem uma reflexão sobre metas e propósito de vida^(11)^, aspecto também inerente à intervenção proposta no presente estudo.

Recomenda-se, para aprimoramentos futuros, a incorporação de metodologias coproduzidas^(20)^, de abordagens baseadas nos pontos fortes^(17)^ e de estratégias fundamentadas na lógica do *peer support* ^(41,42)^, as quais podem ampliar o engajamento das participantes e potencializar os efeitos terapêuticos das ações voltadas à promoção da saúde mental no contexto prisional.

Para a implementação e sustentabilidade de tais intervenções, destaca-se o papel central da enfermagem, especialmente da enfermagem psiquiátrica, cuja prática é historicamente fundamentada no relacionamento interpessoal, na comunicação terapêutica e no acolhimento empático. Além disso, o cuidado é orientado por uma perspectiva integral e relacional, na qual o profissional atua junto ao indivíduo para favorecer a construção de estratégias de enfrentamento e o fortalecimento da autonomia. Esse processo envolve a consideração das singularidades de cada sujeito, suas condições de vida e os recursos disponíveis em diferentes contextos sociais e econômicos, articulando-se continuamente com os demais membros da equipe multiprofissional^(14)^.

Estudos recentes reforçam o protagonismo do enfermeiro na coordenação do cuidado entre os serviços de saúde e o sistema prisional^(13)^, bem como na condução de intervenções voltadas à promoção da saúde mental e à redução do estigma associado ao sofrimento psíquico em contextos de privação de liberdade^(15)^. Nesse sentido, o caráter inovador da presente intervenção reside, em parte, no fato de ter sido implementada por uma enfermeira, demonstrando a capacidade da enfermagem psiquiátrica de conduzir práticas terapêuticas estruturadas e efetivas capazes de promover espaços de escuta, reflexão e ressignificação da experiência de privação de liberdade.

### Limitações do estudo

As limitações do estudo são sua modalidade piloto do tipo prée pós-teste sem grupo controle que comportou uma amostra pequena e propiciou análises apenas do tipo não paramétrico, não sendo possível inferir causalidade entre os aspectos observados. Ainda, a restrição em relação à inclusão de outras participantes que pudessem inclusive ter problemas de comportamento limitou a possibilidade avaliativa da intervenção, além de que as melhorias apresentadas pelas participantes podem estar relacionadas a outros fatores do contexto prisional que não foram consideradas no presente estudo. Apesar das limitações, destaca-se a relevância clínica dos achados do presente estudo. Sugere-se a realização de pesquisas futuras que avaliem os resultados de tal intervenção em grupos diagnósticos específicos, com amostras maiores, melhor tamanho de efeito e delineamentos mais robustos, a fim de proporcionar parâmetros mais precisos sobre o seu potencial terapêutico.

### Contribuições para a área da enfermagem

Os achados do estudo oferecem diversas contribuições relevantes para a prática da enfermagem, especialmente no contexto do cuidado em saúde mental no sistema prisional feminino. Primeiramente, evidenciou-se o potencial de intervenções breves conduzidas por enfermeiras, uma vez que, mesmo com as limitações do estudo, observou-se uma redução significativa nos escores de tensão e depressão, sugerindo que abordagens baseadas em TCCs e no aconselhamento em enfermagem psiquiátrica podem ser viáveis e eficazes nesse contexto. Além disso, as tarefas propostas durante a intervenção, com o “Passatempo sentiMental”, demonstraram potencial de continuidade autônoma, permitindo que as participantes refletissem sobre suas experiências e emoções fora das sessões, promovendo o protagonismo das internas em seu processo de cuidado.

## CONCLUSÕES

Em relação à intervenção realizada, apesar de ser um protocolo de estudo e os resultados estarem relacionados apenas à aplicação desse piloto, foi possível observar melhora em todas as medidas avaliadas, com significância estatística nos itens depressão e tensão da BRUMS. Também se observou decréscimo nos aspectos somáticos, comportamentais e emocionais dos sintomas de transtorno mental comum, sugerindo o potencial da intervenção proposta como uma opção terapêutica não farmacológica para os enfermeiros que atuam no sistema prisional.

## Data Availability

Os dados de pesquisa só estão disponíveis mediante solicitação.
